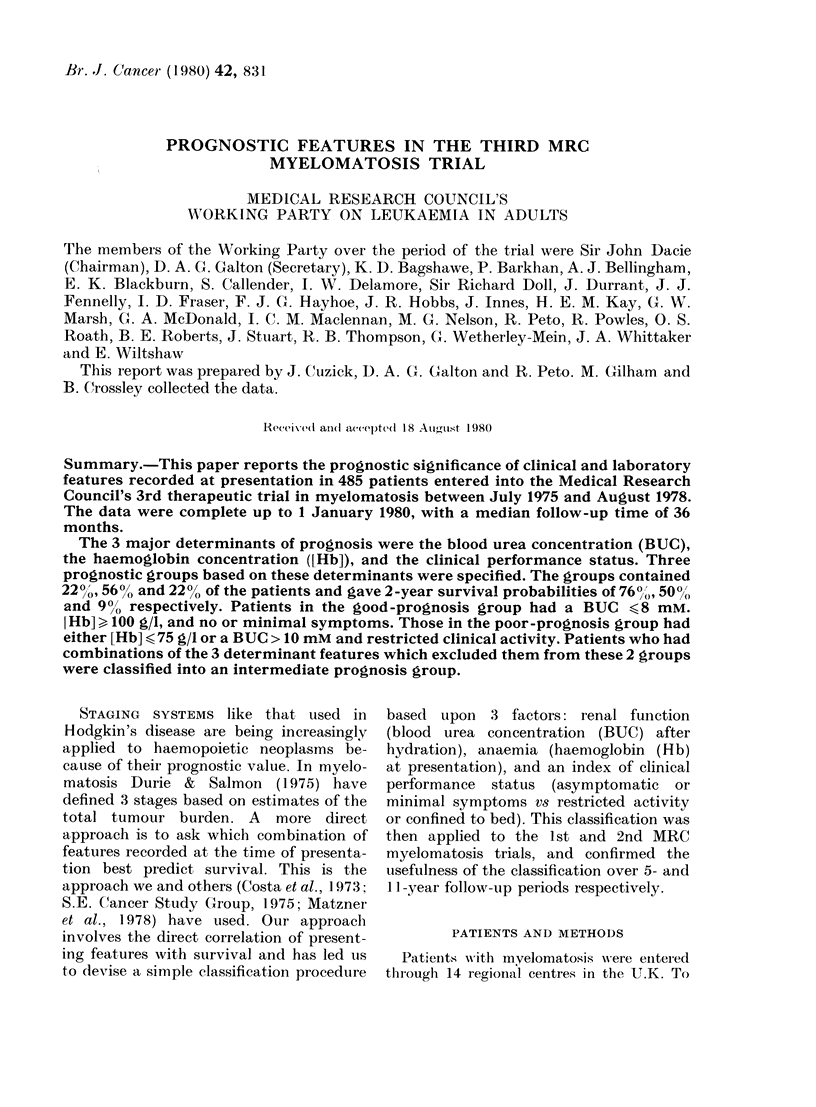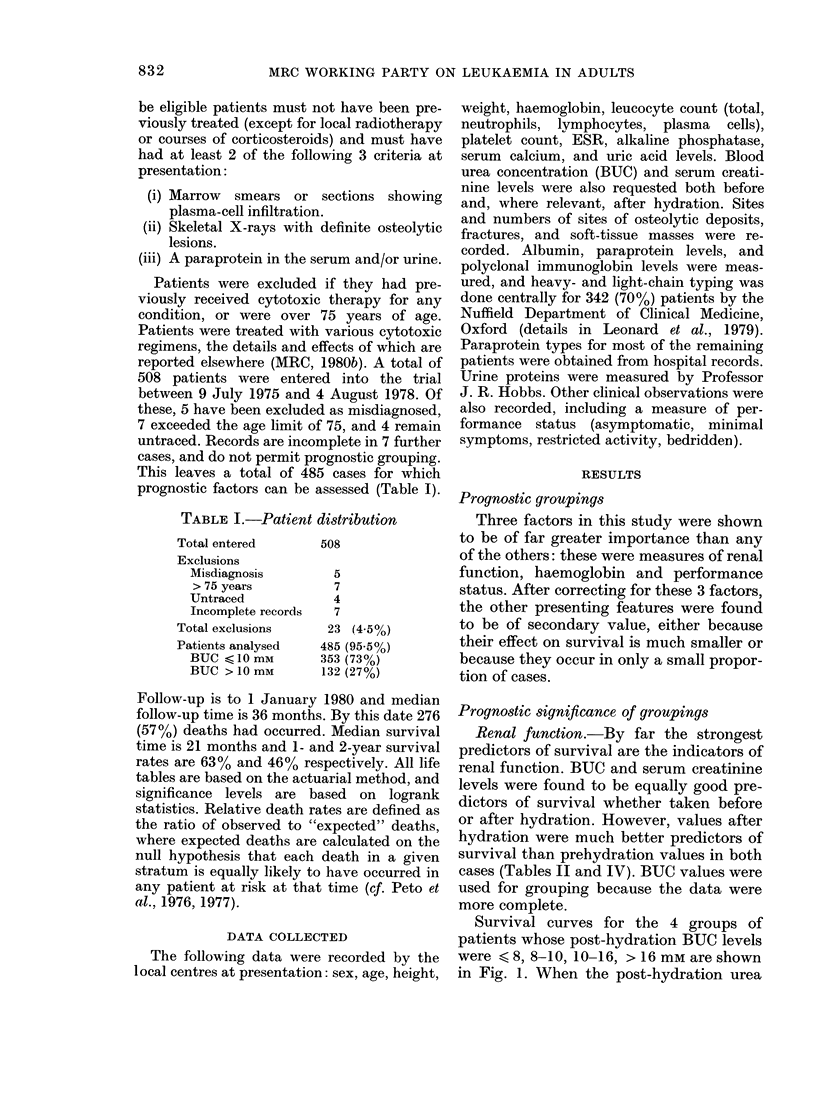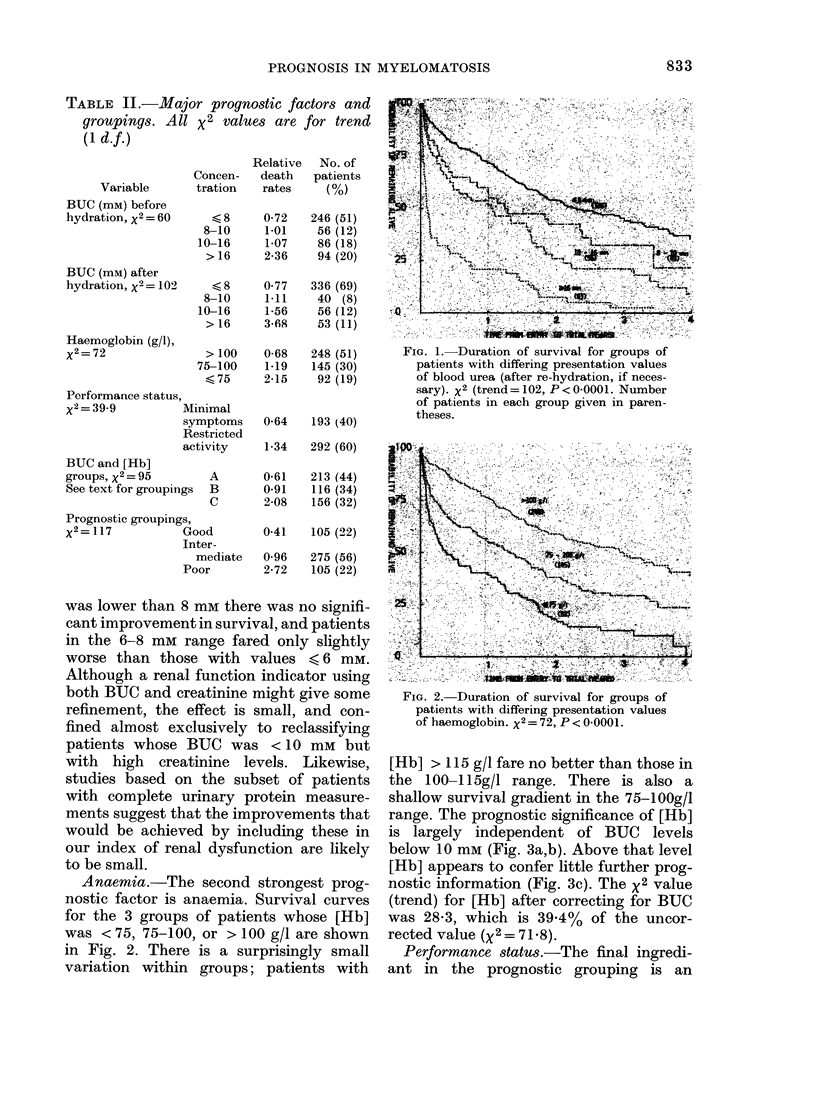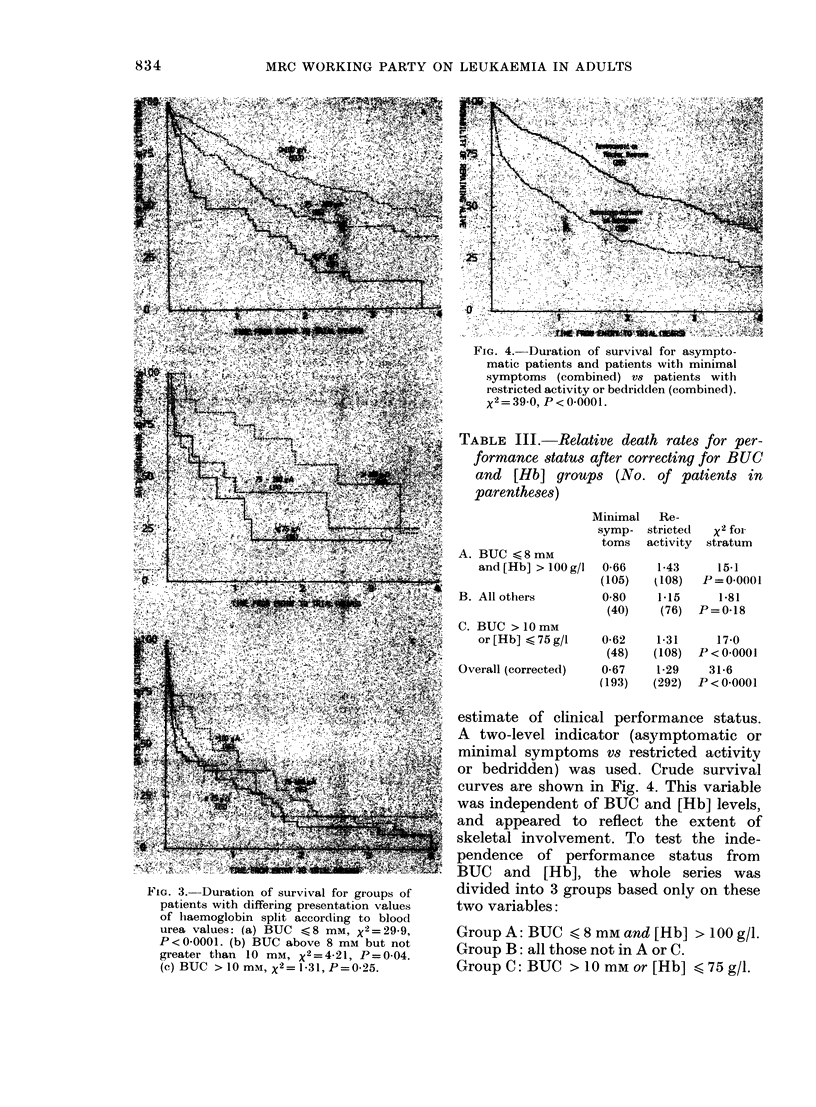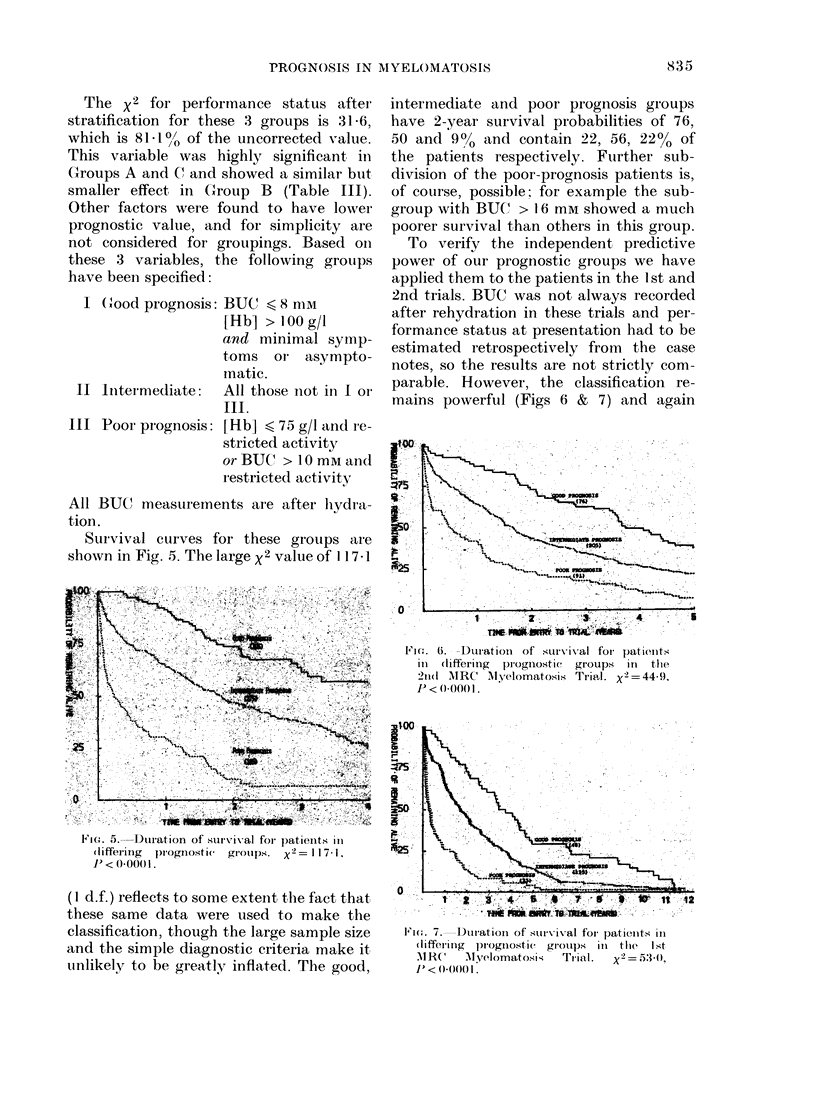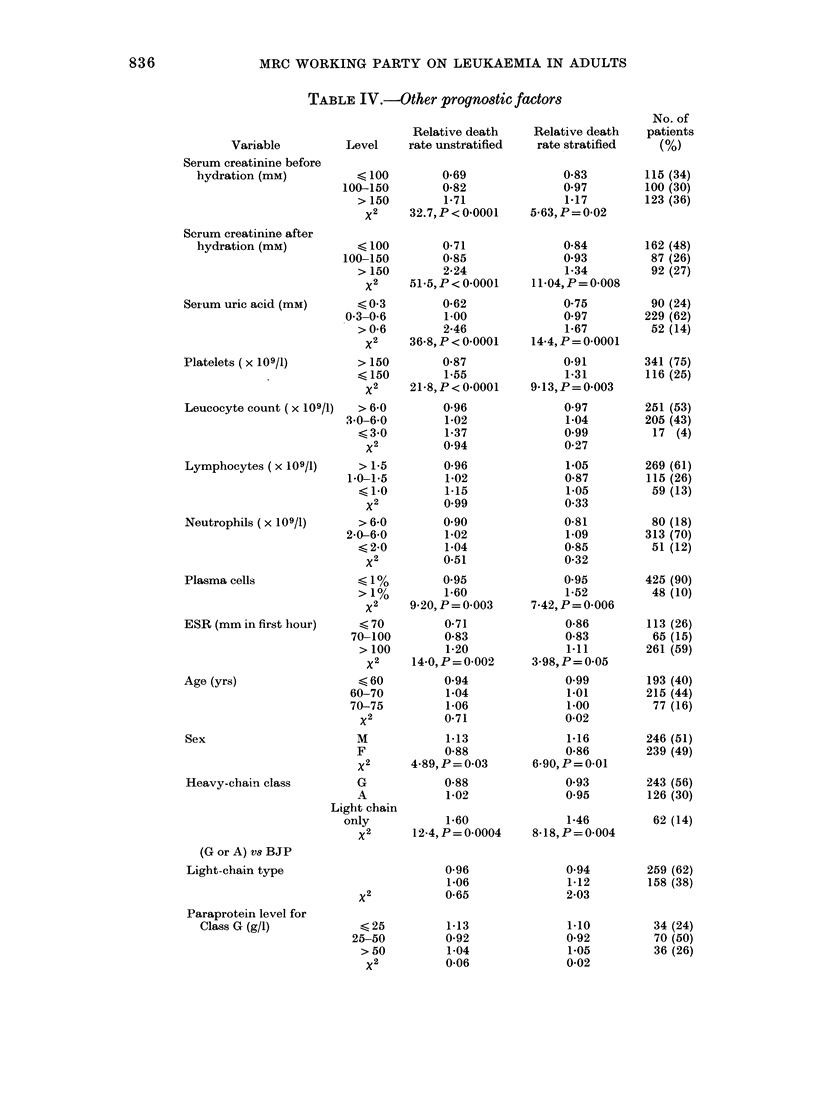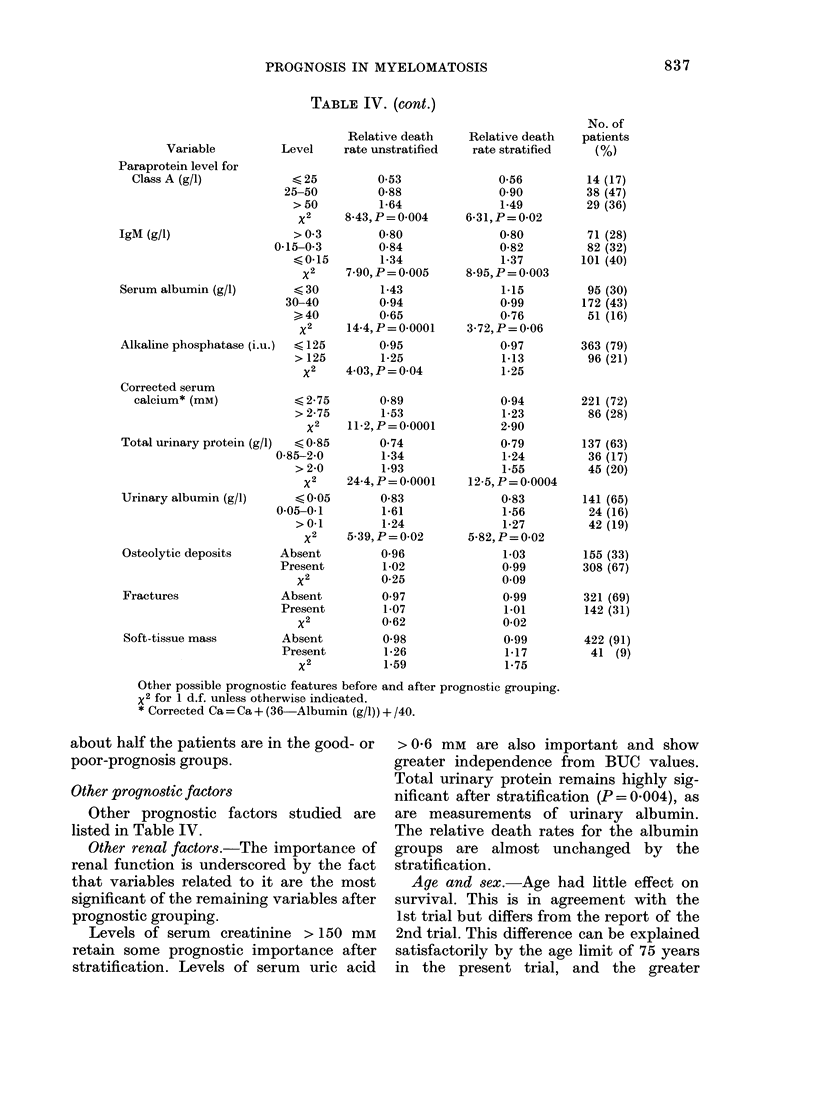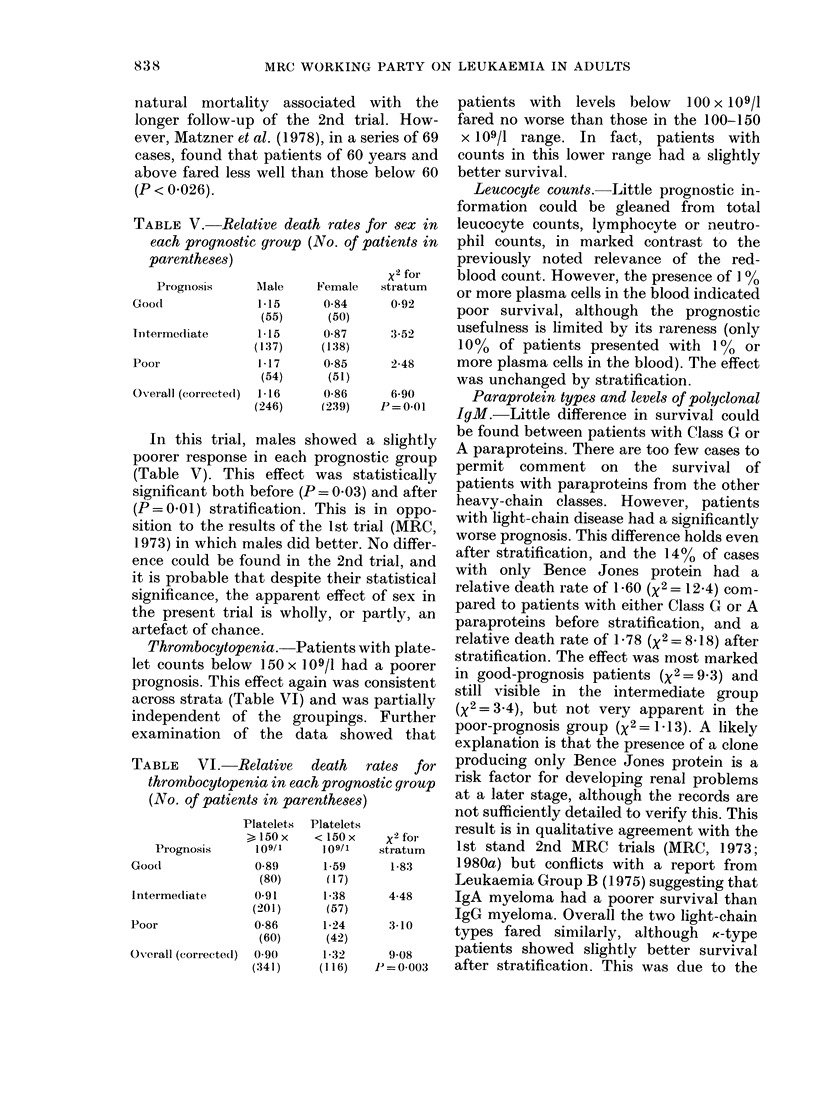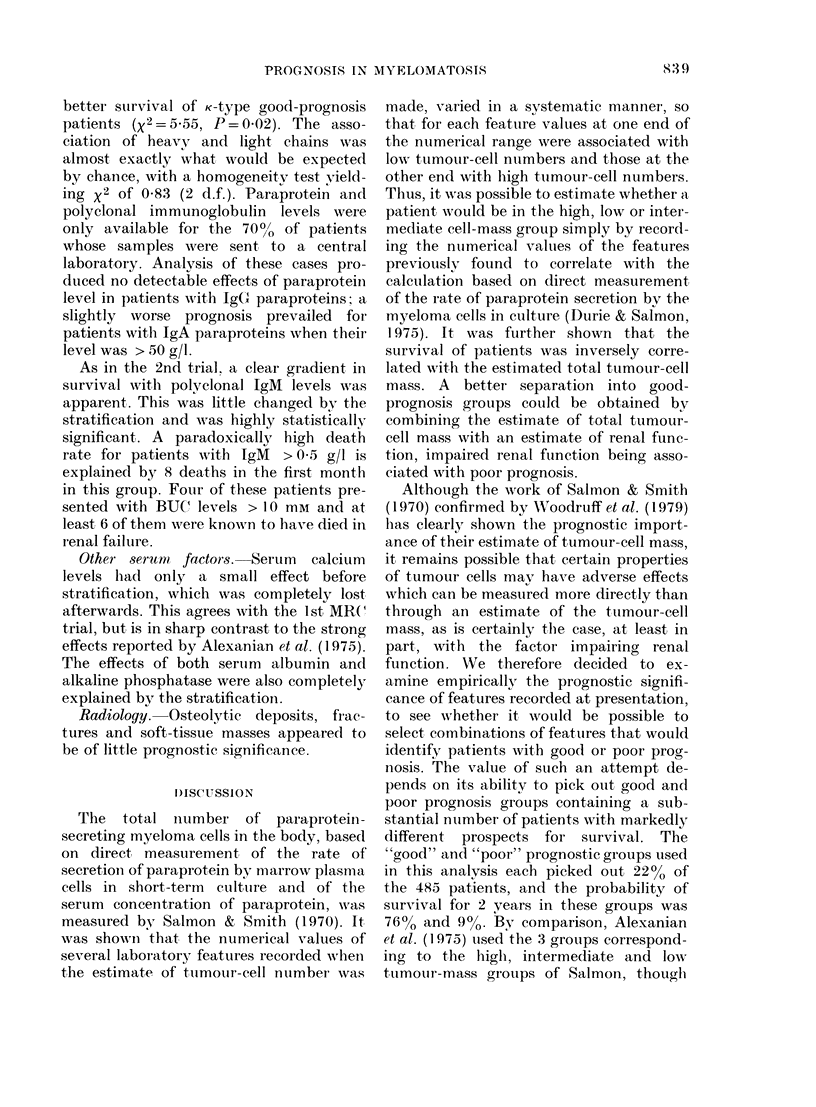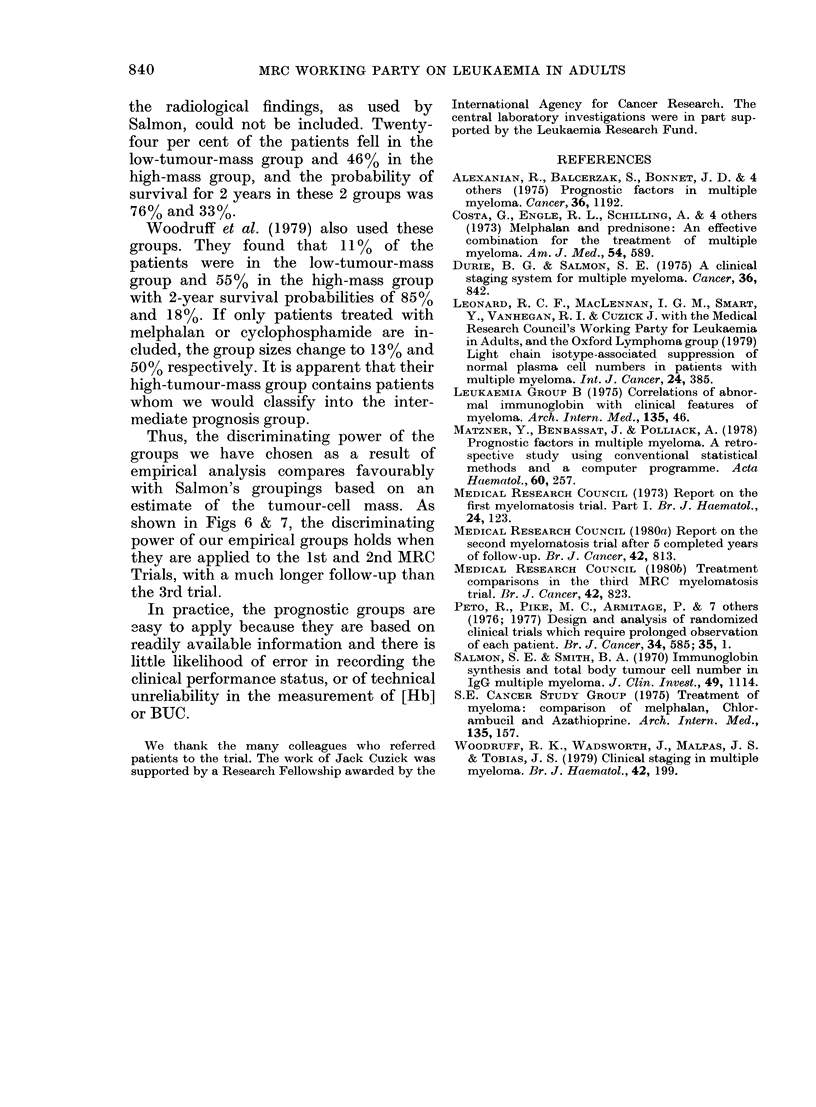# Prognostic features in the third MRC myelomatosis trial. Medical Research Council's Working Party on Leukaemia in Adults.

**DOI:** 10.1038/bjc.1980.330

**Published:** 1980-12

**Authors:** 

## Abstract

This paper reports the prognostic significance of clinical and laboratory features recorded at presentation in 485 patients entered into the Medical Research Council's 3rd therapeutic trial in myelomatosis between July 1975 and August 1978. The data were complete up to 1 January 1980, with a median follow-up time of 36 months. The 3 major determinants of prognosis were the blood urea concentration (BUC), the haemoglobin concentration ([Hb]), and the clinical performance status. Three prognostic groups based on these determinants were specified. The groups contained 22%, 56% and 22% of the patients and gave 2-year survival probabilities of 76%, 50% and 9% respectively. Patients in the good-prognosis group had a BUC less than or equal to 8 mM. [Hb] greater than or equal to 100 g/l, and no or minimal symptoms. Those in the poor-prognosis group had either [Hb] less than or equal to 75 g/l or a BUC greater than 10 mM and restricted clinical activity. Patients who had combinations of the 3 determinant features which excluded them from these 2 groups were classified into an intermediate prognosis group.


*Br. J. Cancer* (1980) **42**, 831

### PROGNOSTIC FEATURES IN THE THIRD MRC MYELOMATOSIS TRIAL

MEDICAL RESEARCH COUNCIL'S
WORKING PARTY ON LEUKAEMIA IN ADULTS

The members of the Working Party over the period of the trial were Sir John Dacie (Chairman), D. A. G. Galton (Secretary), K. D. Bagshawe, P. Barkhan, A. J. Bellingham, E. K. Blackburn, S. Callender, I. W. Delamore, Sir Richard Doll, J. Durrant, J. J. Fennelly, I. D. Fraser, F. J. G. Hayhoe, J. R. Hobbs, J. Innes, H. E. M. Kay, G. W. Marsh, G. A. McDonald, I. C. M. Maclennan, M. G. Nelson, R. Peto, R. Powles, O. S. Roath, B. E. Roberts, J. Stuart, R. B. Thompson, G. Wetherley-Mein, J. A. Whittaker and E. Wiltshaw

This report was prepared by J. Cuzick, D. A. G. Galton and R. Peto. M. Gilham and B. Crossley collected the data.

Received and accepted 18 August 1980

**Summary.—This paper reports the prognostic significance of clinical and laboratory features recorded at presentation in 485 patients entered into the Medical Research Council's 3rd therapeutic trial in myelomatosis between July 1975 and August 1978. The data were complete up to 1 January 1980, with a median follow-up time of 36 months.**

**The 3 major determinants of prognosis were the blood urea concentration (BUC), the haemoglobin concentration ([Hb]), and the clinical performance status. Three prognostic groups based on these determinants were specified. The groups contained 22%, 56% and 22% of the patients and gave 2-year survival probabilities of 76%, 50% and 9% respectively. Patients in the good-prognosis group had a BUC $\leqslant$8 mM. [Hb]$\geqslant$100 g/l, and no or minimal symptoms. Those in the poor-prognosis group had either [Hb]$\leqslant$75 g/l or a BUC > 10 mM and restricted clinical activity. Patients who had combinations of the 3 determinant features which excluded them from these 2 groups were classified into an intermediate prognosis group.**

STAGING SYSTEMS like that used in Hodgkin's disease are being increasingly applied to haemopoietic neoplasms because of their prognostic value. In myelomatosis Durie & Salmon (1975) have defined 3 stages based on estimates of the total tumour burden. A more direct approach is to ask which combination of features recorded at the time of presentation best predict survival. This is the approach we and others (Costa *et al.*, 1973; S.E. Cancer Study Group, 1975; Matzner *et al.*, 1978) have used. Our approach involves the direct correlation of presenting features with survival and has led us to devise a simple classification procedure based upon 3 factors: renal function (blood urea concentration (BUC) after hydration), anaemia (haemoglobin (Hb) at presentation), and an index of clinical performance status (asymptomatic or minimal symptoms *vs* restricted activity or confined to bed). This classification was then applied to the 1st and 2nd MRC myelomatosis trials, and confirmed the usefulness of the classification over 5- and 11-year follow-up periods respectively.

### PATIENTS AND METHODS

Patients with myelomatosis were entered through 14 regional centres in the U.K. To be eligible patients must not have been previously treated (except for local radiotherapy or courses of corticosteroids) and must have had at least 2 of the following 3 criteria at presentation:

(i) Marrow smears or sections showing plasma-cell infiltration.
(ii) Skeletal X-rays with definite osteolytic lesions.
(iii) A paraprotein in the serum and/or urine.

Patients were excluded if they had previously received cytotoxic therapy for any condition, or were over 75 years of age. Patients were treated with various cytotoxic regimens, the details and effects of which are reported elsewhere (MRC, 1980*b*). A total of 508 patients were entered into the trial between 9 July 1975 and 4 August 1978. Of these, 5 have been excluded as misdiagnosed, 7 exceeded the age limit of 75, and 4 remain untraced. Records are incomplete in 7 further cases, and do not permit prognostic grouping. This leaves a total of 485 cases for which prognostic factors can be assessed (Table I).

TABLE I.—*Patient distribution*

| | | |
|---|---|---|
| Total entered | 508 | |
| Exclusions | | |
| Misdiagnosis | 5 | |
| > 75 years | 7 | |
| Untraced | 4 | |
| Incomplete records | 7 | |
| Total exclusions | 23 | (4·5%) |
| Patients analysed | 485 | (95·5%) |
| BUC ≤ 10 mM | 353 | (73%) |
| BUC > 10 mM | 132 | (27%) |

Follow-up is to 1 January 1980 and median follow-up time is 36 months. By this date 276 (57%) deaths had occurred. Median survival time is 21 months and 1- and 2-year survival rates are 63% and 46% respectively. All life tables are based on the actuarial method, and significance levels are based on logrank statistics. Relative death rates are defined as the ratio of observed to "expected" deaths, where expected deaths are calculated on the null hypothesis that each death in a given stratum is equally likely to have occurred in any patient at risk at that time (*cf.* Peto *et al.*, 1976, 1977).

### DATA COLLECTED

The following data were recorded by the local centres at presentation: sex, age, height, weight, haemoglobin, leucocyte count (total, neutrophils, lymphocytes, plasma cells), platelet count, ESR, alkaline phosphatase, serum calcium, and uric acid levels. Blood urea concentration (BUC) and serum creatinine levels were also requested both before and, where relevant, after hydration. Sites and numbers of sites of osteolytic deposits, fractures, and soft-tissue masses were recorded. Albumin, paraprotein levels, and polyclonal immunoglobin levels were measured, and heavy- and light-chain typing was done centrally for 342 (70%) patients by the Nuffield Department of Clinical Medicine, Oxford (details in Leonard *et al.*, 1979). Paraprotein types for most of the remaining patients were obtained from hospital records. Urine proteins were measured by Professor J. R. Hobbs. Other clinical observations were also recorded, including a measure of performance status (asymptomatic, minimal symptoms, restricted activity, bedridden).

### RESULTS

### *Prognostic groupings*

Three factors in this study were shown to be of far greater importance than any of the others: these were measures of renal function, haemoglobin and performance status. After correcting for these 3 factors, the other presenting features were found to be of secondary value, either because their effect on survival is much smaller or because they occur in only a small proportion of cases.

### *Prognostic significance of groupings*

*Renal function.*—By far the strongest predictors of survival are the indicators of renal function. BUC and serum creatinine levels were found to be equally good predictors of survival whether taken before or after hydration. However, values after hydration were much better predictors of survival than prehydration values in both cases (Tables II and IV). BUC values were used for grouping because the data were more complete.

Survival curves for the 4 groups of patients whose post-hydration BUC levels were ≤ 8, 8–10, 10–16, > 16 mM are shown in Fig. 1. When the post-hydration urea

TABLE II.—*Major prognostic factors and groupings. All* $\chi^2$ *values are for trend (1 d.f.)*

| Variable | Concentration | Relative death rates | No. of patients (%) |
|---|---|---|---|
| BUC (mM) before hydration, $\chi^2=60$ | ≤8 | 0·72 | 246 (51) |
| | 8–10 | 1·01 | 56 (12) |
| | 10–16 | 1·07 | 86 (18) |
| | >16 | 2·36 | 94 (20) |
| BUC (mM) after hydration, $\chi^2=102$ | ≤8 | 0·77 | 336 (69) |
| | 8–10 | 1·11 | 40 (8) |
| | 10–16 | 1·56 | 56 (12) |
| | >16 | 3·68 | 53 (11) |
| Haemoglobin (g/l), $\chi^2=72$ | >100 | 0·68 | 248 (51) |
| | 75–100 | 1·19 | 145 (30) |
| | ≤75 | 2·15 | 92 (19) |
| Performance status, $\chi^2=39{\cdot}9$ | Minimal symptoms | 0·64 | 193 (40) |
| | Restricted activity | 1·34 | 292 (60) |
| BUC and [Hb] groups, $\chi^2=95$ | A | 0·61 | 213 (44) |
| See text for groupings | B | 0·91 | 116 (34) |
| | C | 2·08 | 156 (32) |
| Prognostic groupings, $\chi^2=117$ | Good | 0·41 | 105 (22) |
| | Intermediate | 0·96 | 275 (56) |
| | Poor | 2·72 | 105 (22) |

was lower than 8 mM there was no significant improvement in survival, and patients in the 6–8 mM range fared only slightly worse than those with values ≤6 mM. Although a renal function indicator using both BUC and creatinine might give some refinement, the effect is small, and confined almost exclusively to reclassifying patients whose BUC was <10 mM but with high creatinine levels. Likewise, studies based on the subset of patients with complete urinary protein measurements suggest that the improvements that would be achieved by including these in our index of renal dysfunction are likely to be small.

*Anaemia.*—The second strongest prognostic factor is anaemia. Survival curves for the 3 groups of patients whose [Hb] was <75, 75–100, or >100 g/l are shown in Fig. 2. There is a surprisingly small variation within groups; patients with

FIG. 1.—Duration of survival for groups of patients with differing presentation values of blood urea (after re-hydration, if necessary). $\chi^2$ (trend = 102, $P<0{\cdot}0001$. Number of patients in each group given in parentheses.

FIG. 2.—Duration of survival for groups of patients with differing presentation values of haemoglobin. $\chi^2=72$, $P<0{\cdot}0001$.

[Hb] >115 g/l fare no better than those in the 100–115g/l range. There is also a shallow survival gradient in the 75–100g/l range. The prognostic significance of [Hb] is largely independent of BUC levels below 10 mM (Fig. 3a,b). Above that level [Hb] appears to confer little further prognostic information (Fig. 3c). The $\chi^2$ value (trend) for [Hb] after correcting for BUC was 28·3, which is 39·4% of the uncorrected value ($\chi^2=71{\cdot}8$).

*Performance status.*—The final ingredient in the prognostic grouping is an

FIG. 3.—Duration of survival for groups of patients with differing presentation values of haemoglobin split according to blood urea values: (a) BUC ≤8 mM, $\chi^2=29{\cdot}9$, $P<0{\cdot}0001$. (b) BUC above 8 mM but not greater than 10 mM, $\chi^2=4{\cdot}21$, $P=0{\cdot}04$. (c) BUC >10 mM, $\chi^2=1{\cdot}31$, $P=0{\cdot}25$.

FIG. 4.—Duration of survival for asymptomatic patients and patients with minimal symptoms (combined) *vs* patients with restricted activity or bedridden (combined). $\chi^2=39{\cdot}0$, $P<0{\cdot}0001$.

TABLE III.—*Relative death rates for performance status after correcting for BUC and [Hb] groups (No. of patients in parentheses)*

| | Minimal symptoms | Restricted activity | $\chi^2$ for stratum |
|---|---|---|---|
| A. BUC ≤8 mM and [Hb] > 100 g/l | 0·66 (105) | 1·43 (108) | 15·1 $P=0{\cdot}0001$ |
| B. All others | 0·80 (40) | 1·15 (76) | 1·81 $P=0{\cdot}18$ |
| C. BUC >10 mM or [Hb] ≤75 g/l | 0·62 (48) | 1·31 (108) | 17·0 $P<0{\cdot}0001$ |
| Overall (corrected) | 0·67 (193) | 1·29 (292) | 31·6 $P<0{\cdot}0001$ |

estimate of clinical performance status. A two-level indicator (asymptomatic or minimal symptoms *vs* restricted activity or bedridden) was used. Crude survival curves are shown in Fig. 4. This variable was independent of BUC and [Hb] levels, and appeared to reflect the extent of skeletal involvement. To test the independence of performance status from BUC and [Hb], the whole series was divided into 3 groups based only on these two variables:

Group A: BUC ≤ 8 mM *and* [Hb] > 100 g/l.
Group B: all those not in A or C.
Group C: BUC > 10 mM *or* [Hb] ≤ 75 g/l.

The $\chi^2$ for performance status after stratification for these 3 groups is 31·6, which is 81·1% of the uncorrected value. This variable was highly significant in Groups A and C and showed a similar but smaller effect in Group B (Table III). Other factors were found to have lower prognostic value, and for simplicity are not considered for groupings. Based on these 3 variables, the following groups have been specified:

| | | |
|---|---|---|
| I | Good prognosis: | BUC $\leqslant 8$ mM<br>[Hb] $> 100$ g/l<br>*and* minimal symptoms or asymptomatic. |
| II | Intermediate: | All those not in I or III. |
| III | Poor prognosis: | [Hb] $\leqslant 75$ g/l and restricted activity<br>*or* BUC $> 10$ mM and restricted activity |

All BUC measurements are after hydration.

Survival curves for these groups are shown in Fig. 5. The large $\chi^2$ value of 117·1

FIG. 5.—Duration of survival for patients in differing prognostic groups. $\chi^2 = 117 \cdot 1$, $P < 0 \cdot 0001$.

(1 d.f.) reflects to some extent the fact that these same data were used to make the classification, though the large sample size and the simple diagnostic criteria make it unlikely to be greatly inflated. The good, intermediate and poor prognosis groups have 2-year survival probabilities of 76, 50 and 9% and contain 22, 56, 22% of the patients respectively. Further subdivision of the poor-prognosis patients is, of course, possible; for example the subgroup with BUC $> 16$ mM showed a much poorer survival than others in this group.

To verify the independent predictive power of our prognostic groups we have applied them to the patients in the 1st and 2nd trials. BUC was not always recorded after rehydration in these trials and performance status at presentation had to be estimated retrospectively from the case notes, so the results are not strictly comparable. However, the classification remains powerful (Figs 6 & 7) and again

FIG. 6.—Duration of survival for patients in differing prognostic groups in the 2nd MRC Myelomatosis Trial. $\chi^2 = 44 \cdot 9$, $P < 0 \cdot 0001$.

FIG. 7.—Duration of survival for patients in differing prognostic groups in the 1st MRC Myelomatosis Trial. $\chi^2 = 53 \cdot 0$, $P < 0 \cdot 0001$.

TABLE IV.—*Other prognostic factors*

| Variable | Level | Relative death rate unstratified | Relative death rate stratified | No. of patients (%) |
|---|---|---|---|---|
| Serum creatinine before hydration (mM) | ⩽100 | 0·69 | 0·83 | 115 (34) |
| | 100–150 | 0·82 | 0·97 | 100 (30) |
| | >150 | 1·71 | 1·17 | 123 (36) |
| | $\chi^2$ | 32.7, $P<0·0001$ | 5·63, $P=0·02$ | |
| Serum creatinine after hydration (mM) | ⩽100 | 0·71 | 0·84 | 162 (48) |
| | 100–150 | 0·85 | 0·93 | 87 (26) |
| | >150 | 2·24 | 1·34 | 92 (27) |
| | $\chi^2$ | 51·5, $P<0·0001$ | 11·04, $P=0·008$ | |
| Serum uric acid (mM) | ⩽0·3 | 0·62 | 0·75 | 90 (24) |
| | 0·3–0·6 | 1·00 | 0·97 | 229 (62) |
| | >0·6 | 2·46 | 1·67 | 52 (14) |
| | $\chi^2$ | 36·8, $P<0·0001$ | 14·4, $P=0·0001$ | |
| Platelets (×10⁹/l) | >150 | 0·87 | 0·91 | 341 (75) |
| | ⩽150 | 1·55 | 1·31 | 116 (25) |
| | $\chi^2$ | 21·8, $P<0·0001$ | 9·13, $P=0·003$ | |
| Leucocyte count (×10⁹/l) | >6·0 | 0·96 | 0·97 | 251 (53) |
| | 3·0–6·0 | 1·02 | 1·04 | 205 (43) |
| | ⩽3·0 | 1·37 | 0·99 | 17 (4) |
| | $\chi^2$ | 0·94 | 0·27 | |
| Lymphocytes (×10⁹/l) | >1·5 | 0·96 | 1·05 | 269 (61) |
| | 1·0–1·5 | 1·02 | 0·87 | 115 (26) |
| | ⩽1·0 | 1·15 | 1·05 | 59 (13) |
| | $\chi^2$ | 0·99 | 0·33 | |
| Neutrophils (×10⁹/l) | >6·0 | 0·90 | 0·81 | 80 (18) |
| | 2·0–6·0 | 1·02 | 1·09 | 313 (70) |
| | ⩽2·0 | 1·04 | 0·85 | 51 (12) |
| | $\chi^2$ | 0·51 | 0·32 | |
| Plasma cells | ⩽1% | 0·95 | 0·95 | 425 (90) |
| | >1% | 1·60 | 1·52 | 48 (10) |
| | $\chi^2$ | 9·20, $P=0·003$ | 7·42, $P=0·006$ | |
| ESR (mm in first hour) | ⩽70 | 0·71 | 0·86 | 113 (26) |
| | 70–100 | 0·83 | 0·83 | 65 (15) |
| | >100 | 1·20 | 1·11 | 261 (59) |
| | $\chi^2$ | 14·0, $P=0·002$ | 3·98, $P=0·05$ | |
| Age (yrs) | ⩽60 | 0·94 | 0·99 | 193 (40) |
| | 60–70 | 1·04 | 1·01 | 215 (44) |
| | 70–75 | 1·06 | 1·00 | 77 (16) |
| | $\chi^2$ | 0·71 | 0·02 | |
| Sex | M | 1·13 | 1·16 | 246 (51) |
| | F | 0·88 | 0·86 | 239 (49) |
| | $\chi^2$ | 4·89, $P=0·03$ | 6·90, $P=0·01$ | |
| Heavy-chain class | G | 0·88 | 0·93 | 243 (56) |
| | A | 1·02 | 0·95 | 126 (30) |
| | Light chain only | 1·60 | 1·46 | 62 (14) |
| | $\chi^2$ | 12·4, $P=0·0004$ | 8·18, $P=0·004$ | |
| (G or A) *vs* BJP | | | | |
| Light-chain type | | 0·96 | 0·94 | 259 (62) |
| | | 1·06 | 1·12 | 158 (38) |
| | $\chi^2$ | 0·65 | 2·03 | |
| Paraprotein level for Class G (g/l) | ⩽25 | 1·13 | 1·10 | 34 (24) |
| | 25–50 | 0·92 | 0·92 | 70 (50) |
| | >50 | 1·04 | 1·05 | 36 (26) |
| | $\chi^2$ | 0·06 | 0·02 | |

TABLE IV. (*cont.*)

| Variable | Level | Relative death rate unstratified | Relative death rate stratified | No. of patients (%) |
|---|---|---|---|---|
| Paraprotein level for Class A (g/l) | $\leqslant 25$ | 0·53 | 0·56 | 14 (17) |
| | 25–50 | 0·88 | 0·90 | 38 (47) |
| | $>50$ | 1·64 | 1·49 | 29 (36) |
| | $\chi^2$ | 8·43, $P=0·004$ | 6·31, $P=0·02$ | |
| IgM (g/l) | $>0·3$ | 0·80 | 0·80 | 71 (28) |
| | 0·15–0·3 | 0·84 | 0·82 | 82 (32) |
| | $\leqslant 0·15$ | 1·34 | 1·37 | 101 (40) |
| | $\chi^2$ | 7·90, $P=0·005$ | 8·95, $P=0·003$ | |
| Serum albumin (g/l) | $\leqslant 30$ | 1·43 | 1·15 | 95 (30) |
| | 30–40 | 0·94 | 0·99 | 172 (43) |
| | $\geqslant 40$ | 0·65 | 0·76 | 51 (16) |
| | $\chi^2$ | 14·4, $P=0·0001$ | 3·72, $P=0·06$ | |
| Alkaline phosphatase (i.u.) | $\leqslant 125$ | 0·95 | 0·97 | 363 (79) |
| | $>125$ | 1·25 | 1·13 | 96 (21) |
| | $\chi^2$ | 4·03, $P=0·04$ | 1·25 | |
| Corrected serum calcium* (mM) | $\leqslant 2·75$ | 0·89 | 0·94 | 221 (72) |
| | $>2·75$ | 1·53 | 1·23 | 86 (28) |
| | $\chi^2$ | 11·2, $P=0·0001$ | 2·90 | |
| Total urinary protein (g/l) | $\leqslant 0·85$ | 0·74 | 0·79 | 137 (63) |
| | 0·85–2·0 | 1·34 | 1·24 | 36 (17) |
| | $>2·0$ | 1·93 | 1·55 | 45 (20) |
| | $\chi^2$ | 24·4, $P=0·0001$ | 12·5, $P=0·0004$ | |
| Urinary albumin (g/l) | $\leqslant 0·05$ | 0·83 | 0·83 | 141 (65) |
| | 0·05–0·1 | 1·61 | 1·56 | 24 (16) |
| | $>0·1$ | 1·24 | 1·27 | 42 (19) |
| | $\chi^2$ | 5·39, $P=0·02$ | 5·82, $P=0·02$ | |
| Osteolytic deposits | Absent | 0·96 | 1·03 | 155 (33) |
| | Present | 1·02 | 0·99 | 308 (67) |
| | $\chi^2$ | 0·25 | 0·09 | |
| Fractures | Absent | 0·97 | 0·99 | 321 (69) |
| | Present | 1·07 | 1·01 | 142 (31) |
| | $\chi^2$ | 0·62 | 0·02 | |
| Soft-tissue mass | Absent | 0·98 | 0·99 | 422 (91) |
| | Present | 1·26 | 1·17 | 41 (9) |
| | $\chi^2$ | 1·59 | 1·75 | |

Other possible prognostic features before and after prognostic grouping.
$\chi^2$ for 1 d.f. unless otherwise indicated.
* Corrected Ca = Ca + (36—Albumin (g/l)) + /40.

about half the patients are in the good- or poor-prognosis groups.

*Other prognostic factors*

Other prognostic factors studied are listed in Table IV.

*Other renal factors.*—The importance of renal function is underscored by the fact that variables related to it are the most significant of the remaining variables after prognostic grouping.

Levels of serum creatinine > 150 mM retain some prognostic importance after stratification. Levels of serum uric acid > 0·6 mM are also important and show greater independence from BUC values. Total urinary protein remains highly significant after stratification ($P=0·004$), as are measurements of urinary albumin. The relative death rates for the albumin groups are almost unchanged by the stratification.

*Age and sex.*—Age had little effect on survival. This is in agreement with the 1st trial but differs from the report of the 2nd trial. This difference can be explained satisfactorily by the age limit of 75 years in the present trial, and the greater natural mortality associated with the longer follow-up of the 2nd trial. However, Matzner *et al.* (1978), in a series of 69 cases, found that patients of 60 years and above fared less well than those below 60 ($P < 0{\cdot}026$).

TABLE V.—*Relative death rates for sex in each prognostic group (No. of patients in parentheses)*

| Prognosis | Male | Female | $\chi^2$ for stratum |
|---|---|---|---|
| Good | 1·15 (55) | 0·84 (50) | 0·92 |
| Intermediate | 1·15 (137) | 0·87 (138) | 3·52 |
| Poor | 1·17 (54) | 0·85 (51) | 2·48 |
| Overall (corrected) | 1·16 (246) | 0·86 (239) | 6·90 $P = 0{\cdot}01$ |

In this trial, males showed a slightly poorer response in each prognostic group (Table V). This effect was statistically significant both before ($P = 0{\cdot}03$) and after ($P = 0{\cdot}01$) stratification. This is in opposition to the results of the 1st trial (MRC, 1973) in which males did better. No difference could be found in the 2nd trial, and it is probable that despite their statistical significance, the apparent effect of sex in the present trial is wholly, or partly, an artefact of chance.

*Thrombocytopenia*.—Patients with platelet counts below $150 \times 10^9$/l had a poorer prognosis. This effect again was consistent across strata (Table VI) and was partially independent of the groupings. Further examination of the data showed that

TABLE VI.—*Relative death rates for thrombocytopenia in each prognostic group (No. of patients in parentheses)*

| Prognosis | Platelets $\geqslant 150 \times 10^9$/l | Platelets $< 150 \times 10^9$/l | $\chi^2$ for stratum |
|---|---|---|---|
| Good | 0·89 (80) | 1·59 (17) | 1·83 |
| Intermediate | 0·91 (201) | 1·38 (57) | 4·48 |
| Poor | 0·86 (60) | 1·24 (42) | 3·10 |
| Overall (corrected) | 0·90 (341) | 1·32 (116) | 9·08 $P = 0{\cdot}003$ |

patients with levels below $100 \times 10^9$/l fared no worse than those in the 100–150 $\times 10^9$/l range. In fact, patients with counts in this lower range had a slightly better survival.

*Leucocyte counts*.—Little prognostic information could be gleaned from total leucocyte counts, lymphocyte or neutrophil counts, in marked contrast to the previously noted relevance of the red-blood count. However, the presence of 1% or more plasma cells in the blood indicated poor survival, although the prognostic usefulness is limited by its rareness (only 10% of patients presented with 1% or more plasma cells in the blood). The effect was unchanged by stratification.

*Paraprotein types and levels of polyclonal IgM*.—Little difference in survival could be found between patients with Class G or A paraproteins. There are too few cases to permit comment on the survival of patients with paraproteins from the other heavy-chain classes. However, patients with light-chain disease had a significantly worse prognosis. This difference holds even after stratification, and the 14% of cases with only Bence Jones protein had a relative death rate of 1·60 ($\chi^2 = 12{\cdot}4$) compared to patients with either Class G or A paraproteins before stratification, and a relative death rate of 1·78 ($\chi^2 = 8{\cdot}18$) after stratification. The effect was most marked in good-prognosis patients ($\chi^2 = 9{\cdot}3$) and still visible in the intermediate group ($\chi^2 = 3{\cdot}4$), but not very apparent in the poor-prognosis group ($\chi^2 = 1{\cdot}13$). A likely explanation is that the presence of a clone producing only Bence Jones protein is a risk factor for developing renal problems at a later stage, although the records are not sufficiently detailed to verify this. This result is in qualitative agreement with the 1st stand 2nd MRC trials (MRC, 1973; 1980*a*) but conflicts with a report from Leukaemia Group B (1975) suggesting that IgA myeloma had a poorer survival than IgG myeloma. Overall the two light-chain types fared similarly, although $\kappa$-type patients showed slightly better survival after stratification. This was due to the better survival of $\kappa$-type good-prognosis patients ($\chi^2 = 5{\cdot}55$, $P = 0{\cdot}02$). The association of heavy and light chains was almost exactly what would be expected by chance, with a homogeneity test yielding $\chi^2$ of 0·83 (2 d.f.). Paraprotein and polyclonal immunoglobulin levels were only available for the 70% of patients whose samples were sent to a central laboratory. Analysis of these cases produced no detectable effects of paraprotein level in patients with IgG paraproteins; a slightly worse prognosis prevailed for patients with IgA paraproteins when their level was > 50 g/l.

As in the 2nd trial, a clear gradient in survival with polyclonal IgM levels was apparent. This was little changed by the stratification and was highly statistically significant. A paradoxically high death rate for patients with IgM > 0·5 g/l is explained by 8 deaths in the first month in this group. Four of these patients presented with BUC levels > 10 mM and at least 6 of them were known to have died in renal failure.

*Other serum factors.*—Serum calcium levels had only a small effect before stratification, which was completely lost afterwards. This agrees with the 1st MRC trial, but is in sharp contrast to the strong effects reported by Alexanian *et al.* (1975). The effects of both serum albumin and alkaline phosphatase were also completely explained by the stratification.

*Radiology.*—Osteolytic deposits, fractures and soft-tissue masses appeared to be of little prognostic significance.

### DISCUSSION

The total number of paraprotein-secreting myeloma cells in the body, based on direct measurement of the rate of secretion of paraprotein by marrow plasma cells in short-term culture and of the serum concentration of paraprotein, was measured by Salmon & Smith (1970). It was shown that the numerical values of several laboratory features recorded when the estimate of tumour-cell number was made, varied in a systematic manner, so that for each feature values at one end of the numerical range were associated with low tumour-cell numbers and those at the other end with high tumour-cell numbers. Thus, it was possible to estimate whether a patient would be in the high, low or intermediate cell-mass group simply by recording the numerical values of the features previously found to correlate with the calculation based on direct measurement of the rate of paraprotein secretion by the myeloma cells in culture (Durie & Salmon, 1975). It was further shown that the survival of patients was inversely correlated with the estimated total tumour-cell mass. A better separation into good-prognosis groups could be obtained by combining the estimate of total tumour-cell mass with an estimate of renal function, impaired renal function being associated with poor prognosis.

Although the work of Salmon & Smith (1970) confirmed by Woodruff *et al.* (1979) has clearly shown the prognostic importance of their estimate of tumour-cell mass, it remains possible that certain properties of tumour cells may have adverse effects which can be measured more directly than through an estimate of the tumour-cell mass, as is certainly the case, at least in part, with the factor impairing renal function. We therefore decided to examine empirically the prognostic significance of features recorded at presentation, to see whether it would be possible to select combinations of features that would identify patients with good or poor prognosis. The value of such an attempt depends on its ability to pick out good and poor prognosis groups containing a substantial number of patients with markedly different prospects for survival. The "good" and "poor" prognostic groups used in this analysis each picked out 22% of the 485 patients, and the probability of survival for 2 years in these groups was 76% and 9%. By comparison, Alexanian *et al.* (1975) used the 3 groups corresponding to the high, intermediate and low tumour-mass groups of Salmon, though the radiological findings, as used by Salmon, could not be included. Twenty-four per cent of the patients fell in the low-tumour-mass group and 46% in the high-mass group, and the probability of survival for 2 years in these 2 groups was 76% and 33%.

Woodruff *et al.* (1979) also used these groups. They found that 11% of the patients were in the low-tumour-mass group and 55% in the high-mass group with 2-year survival probabilities of 85% and 18%. If only patients treated with melphalan or cyclophosphamide are included, the group sizes change to 13% and 50% respectively. It is apparent that their high-tumour-mass group contains patients whom we would classify into the intermediate prognosis group.

Thus, the discriminating power of the groups we have chosen as a result of empirical analysis compares favourably with Salmon's groupings based on an estimate of the tumour-cell mass. As shown in Figs 6 & 7, the discriminating power of our empirical groups holds when they are applied to the 1st and 2nd MRC Trials, with a much longer follow-up than the 3rd trial.

In practice, the prognostic groups are easy to apply because they are based on readily available information and there is little likelihood of error in recording the clinical performance status, or of technical unreliability in the measurement of [Hb] or BUC.

We thank the many colleagues who referred patients to the trial. The work of Jack Cuzick was supported by a Research Fellowship awarded by the International Agency for Cancer Research. The central laboratory investigations were in part supported by the Leukaemia Research Fund.

### REFERENCES

Alexanian, R., Balcerzak, S., Bonnet, J. D. & 4 others (1975) Prognostic factors in multiple myeloma. *Cancer*, **36**, 1192.

Costa, G., Engle, R. L., Schilling, A. & 4 others (1973) Melphalan and prednisone: An effective combination for the treatment of multiple myeloma. *Am. J. Med.*, **54**, 589.

Durie, B. G. & Salmon, S. E. (1975) A clinical staging system for multiple myeloma. *Cancer*, **36**, 842.

Leonard, R. C. F., MacLennan, I. G. M., Smart, Y., Vanhegan, R. I. & Cuzick J. with the Medical Research Council's Working Party for Leukaemia in Adults, and the Oxford Lymphoma group (1979) Light chain isotype-associated suppression of normal plasma cell numbers in patients with multiple myeloma. *Int. J. Cancer*, **24**, 385.

Leukaemia Group B (1975) Correlations of abnormal immunoglobin with clinical features of myeloma. *Arch. Intern. Med.*, **135**, 46.

Matzner, Y., Benbassat, J. & Polliack, A. (1978) Prognostic factors in multiple myeloma. A retrospective study using conventional statistical methods and a computer programme. *Acta Haematol.*, **60**, 257.

Medical Research Council (1973) Report on the first myelomatosis trial. Part I. *Br. J. Haematol.*, **24**, 123.

Medical Research Council (1980*a*) Report on the second myelomatosis trial after 5 completed years of follow-up. *Br. J. Cancer*, **42**, 813.

Medical Research Council (1980*b*) Treatment comparisons in the third MRC myelomatosis trial. *Br. J. Cancer*, **42**, 823.

Peto, R., Pike, M. C., Armitage, P. & 7 others (1976; 1977) Design and analysis of randomized clinical trials which require prolonged observation of each patient. *Br. J. Cancer*, **34**, 585; **35**, 1.

Salmon, S. E. & Smith, B. A. (1970) Immunoglobin synthesis and total body tumour cell number in IgG multiple myeloma. *J. Clin. Invest.*, **49**, 1114.

S.E. Cancer Study Group (1975) Treatment of myeloma: comparison of melphalan, Chlorambucil and Azathioprine. *Arch. Intern. Med.*, **135**, 157.

Woodruff, R. K., Wadsworth, J., Malpas, J. S. & Tobias, J. S. (1979) Clinical staging in multiple myeloma. *Br. J. Haematol.*, **42**, 199.